# Plant Co-expression Annotation Resource: a web server for identifying targets for genetically modified crop breeding pipelines

**DOI:** 10.1186/s12859-020-03792-z

**Published:** 2021-02-05

**Authors:** Marcos José Andrade Viana, Adhemar Zerlotini, Mauricio de Alvarenga Mudadu

**Affiliations:** 1 Graduate Program in Bioinformatics, Institute of Biological Sciences, Universidade Federal de Minas Gerais, Belo Horizonte, Minas Gerais 31270-901 Brazil; 2 Embrapa Informática Agropecuária, Campinas, São Paulo 6041 Brazil; 3 Embrapa Milho e Sorgo, Sete Lagoas, Minas Gerais 285 Brazil

**Keywords:** Proteins of unknown function, Annotation, Abiotic stress, Database

## Abstract

The development of genetically modified crops (GM) includes the discovery of candidate genes through bioinformatics analysis using genomics data, gene expression, and others. Proteins of unknown function (PUFs) are interesting targets for GM crops breeding pipelines for the novelty associated with such targets and also to avoid copyright protection. One method of inferring the putative function of PUFs is by relating them to factors of interest such as abiotic stresses using orthology and co-expression networks, in a guilt-by-association manner. In this regard, we have downloaded, analyzed, and processed genomics data of 53 angiosperms, totaling 1,862,010 genes and 2,332,974 RNA. Diamond and InterproScan were used to discover 72,266 PUFs for all organisms. RNA-seq datasets related to abiotic stresses were downloaded from NCBI/GEO. The RNA-seq data was used as input to the LSTrAP software to construct co-expression networks. LSTrAP also created clusters of transcripts with correlated expression, whose members are more probably related to the molecular mechanisms associated with abiotic stresses in the plants. Orthologous groups were created (OrhtoMCL) using all 2,332,974 proteins in order to associate PUFs to abiotic stress-related clusters of co-expression and therefore infer their function in a guilt-by-association manner. A freely available web resource named “Plant Co-expression Annotation Resource” (https://www.machado.cnptia.embrapa.br/plantannot), *Plantannot*, was created to provide indexed queries to search for PUF putatively associated with abiotic stresses. The web interface also allows browsing, querying, and retrieving of public genomics data from 53 plants. We hope *Plantannot* to be useful for researchers trying to obtain novel GM crops resistant to climate change hazards.

## Background

In the last decades, the ability to genetically engineer plants demonstrated the potential to create genetically modified (GM) crops with favorable economic outcomes [1]. The main achievement in this area was the development of improved plants tolerant to herbicide and resistant to insects, although nutritional composition improvements are about to happen [2]. Furthermore, new mechanisms for genome editing are improving the accuracy and speed of genome modifications in plants, such as the CRISPR/CAS system [3, 4].

Regarding to climate change and environmental factors, plants are being genetically modified to become resilient to abiotic stresses, such as drought, high temperature, rising atmospheric CO2, in order to potentially overcome the yield losses due to these factors [5, 6].

Consequently, over the last years, many patent applications for genetically improved crops regarding stress tolerance were filled [8]. Intellectual property rights (IPR) are vastly used by biotechnology enterprises for their GM plants to allow exclusive rights and yield better returns for the high investments in research and development [7]. To avoid selecting patented genes, it’s possible to start researching genes and proteins with no function yet described.

The first phase for creating GM crops is the candidate gene discovery, which relies on bioinformatics analyses of huge volumes of genomics data available on public resources [8, 9]. These proteins of unknown function (PUF) are very prevalent in eukaryotic genomes and may play a role in determining the differences between species [10] and also may be related to resistance to abiotic stresses [11].

The resistance to abiotic stresses is a complex and multigenic trait. Computational analyses related to QTL, GWAS, gene expression and regulatory networks can be employed to identify genes and molecular mechanisms that may play a role in these conditions [12–14], and successful results were already published [6, 15, 16].

It is known that differences in the gene expression patterns, allied to environmental influences, lead to differences in the morphology and phenotype of animals and plants [17]. It is also well established that organs and tissues with the same evolutionary origin have correlated gene expression patterns [18]. To perform molecular comparisons between different species, it’s necessary to focus on genes with the same evolutionary origin and, therefore, with homolog functions, i.e. orthologs [19]. One approach for studying the regulatory functions of a network of genes over different species is to align the co-expression networks using ortholog genes [20].

In the present work, we present a web resource named “Plant co-expression annotation resource” (https://www.machado.cnptia.embrapa.br/plantannot) which uses plant genomics data, RNA sequencing data, orthology, and co-expression networks to enable the identification of PUFs as abiotic stress-related candidates to enter GM crop breeding pipelines.

## Construction and content

### Raw data

Genome data (sequence assembly in FASTA formatted files and annotation in GFF files) for 53 angiosperms (Table 1), including *Glycine max* (Gma), *Zea mays* (Zma), *Arabidopsis thaliana* (Ath), and *Oryza sativa* (Osa), were obtained from Phytozome v12 [21] and one from NCBI (*Boea hygrometrica*). The total number of genes and mRNA stored was 1,862,010 and 2,332,974, respectively, together with their translated proteins.

**Table 1:** Organisms, genome versions, and PUF quantification

Organism	Genome version	PUF quantification					
		ProtocolA	Protocol B	Protocol C	Protocol D	Protocol E	Protocol F
*Amaranthus hypochondriacus*	v1.0	873	3	3	4	0	2
*Amborella trichopoda*	v1.0	52	0	4	0	0	3
*Ananas comosus*	v3	1790	0	7	4	0	3
*Aquilegia coerulea*	v3.1	2214	10	38	2	0	25
*Arabidopsis halleri*	v1.1	362	0	13	7	0	8
*Arabidopsis lyrata*	v2.1	609	0	4	4	0	3
*Arabidopsis thaliana*	TAIR10	322	0	150	17	0	128
*Boea hygrometrica*	GCA_001598015.1	37	0	2	0	0	0
*Boechera stricta*	v1.2	557	4	14	18	0	10
*Brachypodium distachyon*	v3.1	2018	2	73	6	0	49
*Brachypodium stacei*	v1.1	1060	1	41	2	1	33
*Brassica oleracea capitata*	V1.0	390	0	11	2	0	0
*Brassica rapa*	FPsc	565	1	21	7	0	13
*Capsella grandiflora*	v1.1	202	0	14	9	0	9
*Capsella rubella*	v1.0	2	0	10	0	0	10
*Carica papaya*	ASGPBv0.4	3333	0	0	5	0	0
*Citrus clementenina*	v1.0	7	0	24	0	0	20
*Citrus sinensis*	v1.1	5	0	27	1	0	23
*Cucumis sativus*	v1.0	995	0	20	5	0	18
*Daucus carota*	v2.0	8	0	0	0	0	0
*Eucalyptus grandis*	v2.0	56	0	23	0	0	21
*Eutrema salsugineum*	v1.0	3	0	8	0	0	8
*Fragaria vesca*	v1.1	3142	20	1	2	0	0
*Glycine max*	Wm82.a2.v1	20	0	103	5	0	98
*Gossypium raimondii*	v2.1	18	0	62	0	0	46
*Kalanchoe fedtschenkoi*	v1.1	1933	14	53	5	1	40
*Kalanchoe laxiflora*	v1.1	1576	9	99	7	1	71
*Linum usitatissimum*	v1.0	1542	27	8	7	1	3
*Malus domestica*	v1.0	5025	5	48	7	0	27
*Manihot esculenta*	v6.1	20	0	40	0	0	35
*Medicago truncatula*	Mt4.0v1	229	0	50	0	0	37
*Mimulus guttatus*	v2.0	715	2	36	9	0	27
*Musa acuminata*	v1	3759	2	2	11	0	0
*Oropetium thomaeum*	v1.0	2551	8	7	10	1	4
*Oryza sativa*	v7_JGI	709	0	17	82	0	17
*Panicum hallii*	v2.0	22	0	63	2	0	45
*Panicum virgatum*	v1.1	10,211	6	117	31	1	59
*Phaseolus vulgaris*	v2.1	123	0	36	5	0	35
*Populus trichocarpa*	v3.0	1466	0	124	8	0	94
*Prunus persica*	v2.1	16	0	42	2	0	34
*Ricinus communis*	v0.1	18	0	0	1	0	0
*Salix purpurea*	v1.0	1539	0	0	10	0	0
*Setaria italica*	v2.2	1492	1	59	0	1	38
*Setaria viridis*	v1.1	1896	1	64	1	1	40
*Solanum lycopersicum*	iTAG2.4	2694	0	1	1	0	0
*Solanum tuberosum*	v4.03	3353	2265	3303	4	4	887
*Sorghum bicolor*	v3.1.1	14	0	18	0	0	11
*Spirodela polyrhiza*	v2	1104	13	17	11	0	8
*Theobroma cacao*	v1.1	151	4	1448	0	0	25
*Trifolium pratense*	v2	1630	6	12	8	0	10
*Vitis vinifera*	Genoscope.12X	123	1	1	0	0	0
*Zea mays*	284_AGPv3	9674	3	67	1042	1	60
*Zostera marina*	v2.2	41	1	164	0	0	143
Total	53	72,266	2409	6569	1364	13	2280

RNA-seq data related to abiotic stresses (heat, drought, dehydration, and osmotic stress) were downloaded from NCBI/GEO in a total of 17 different GEO Series, 53 GEO Samples and 60 SRA short read files only for Gma, Zma, Gma and Ath (Table 2). The data was obtained by searching GEO datasets for the given organisms using the keywords “stress” and filtering the study type by "Expression profiling by high throughput sequencing". The raw reads, corresponding to the GEO Samples, were obtained from NCBI/SRA automatically using the sratoolkit v2.9.2 [22].

**Table 2:** GEO experiments, GEO samples, and SRA identifiers used to obtain RNA-seq data

Organism	GEO series	GEO samples	SRA	Condition	Tissue	Date
*Arabidopsis thaliana*	GSE85653	GSM2280286	SRR4033018	Heat stress rep1	Leaves	May-30-2018
*Arabidopsis thaliana*	GSE85653	GSM2280287	SRR4033019	Heat stress rep2	Leaves	May-30-2018
*Arabidopsis thaliana*	GSE85653	GSM2280288	SRR4033020	Heat stress rep3	Leaves	May-30-2018
*Arabidopsis thaliana*	GSE93979	GSM2466002	SRR5196729	WT drought rep1	Leaf	Jun-13-2017
*Arabidopsis thaliana*	GSE93979	GSM2466003	SRR5196730	WT drought rep1	Leaf	Jun-13-2017
*Arabidopsis thaliana*	GSE93420	GSM2453038	SRR5167847	WT_dehydration1	Leaf	Apr-11-2017
*Arabidopsis thaliana*	GSE93420	GSM2453039	SRR5167848	WT_dehydration2	Leaf	Apr-11-2017
*Arabidopsis thaliana*	GSE93420	GSM2453040	SRR5167849	WT_dehydration3	Leaf	Apr-11-2017
*Arabidopsis thaliana*	GSE94015	GSM2467113	SRR5197907	WT RL3h rep1 heat stress (treated at 37 °C for 3 h)	Rosette leaves at flower stages 1–9	Mar-15-2017
*Arabidopsis thaliana*	GSE94015	GSM2467114	SRR5197908	WT RL3h rep2 heat stress (treated at 37 °C for 3 h)	Rosette leaves at flower stages 1–9	Mar-15-2017
*Arabidopsis thaliana*	GSE94015	GSM2467115	SRR5197909	WT RL3h rep3 heat stress (treated at 37 °C for 3 h)	Rosette leaves at flower stages 1-9	Mar-15-2017
*Arabidopsis thaliana*	GSE72806	GSM1872392	SRR2302914	Col h-1R heat stress (44 °C for 1 h)	Leaves	Oct-24-2016
*Arabidopsis thaliana*	GSE72806	GSM1872393	SRR2302915	Col h-2R heat stress (44 °C for 1 h)	Leaves	Oct-24-2016
*Arabidopsis thaliana*	GSE72806	GSM1872394	SRR2302916	Col h-3R heat stress (44 °C for 1 h)	Leaves	Oct-24-2016
*Arabidopsis thaliana*	GSE72806	GSM1872389	SRR2302911	Col s-1R salinity stress	Leaves	Oct-24-2016
*Arabidopsis thaliana*	GSE72806	GSM1872390	SRR2302912	Col s-2R salinity stress	Leaves	Oct-24-2016
*Arabidopsis thaliana*	GSE72806	GSM1872391	SRR2302913	Col s-3R salinity stress	Leaves	Oct-24-2016
*Oryza sativa*	GSE101734	GSM2714235	SRR5856930	Salt	Seedling leaf	Jul-22-2017
*Oryza sativa*	GSE101734	GSM2714236	SRR5856931	Salt	Seedling leaf	Jul-22-2017
*Oryza sativa*	GSE101734	GSM2714237	SRR5856932	Salt	Seedling leaf	Jul-22-2017
*Oryza sativa*	GSE77510	GSM2053502	SRR3140959	Heat stress (45 °C)—12 h	Leaf	Dec-21-2017
*Oryza sativa*	GSE78972	GSM2082859	SRR3209771	Long Day Drought_S3	Leaf	Mar-01-2017
*Oryza sativa*	GSE78972	GSM2082860	SRR3209772	Long Day Drought_S4	Leaf	Mar-01-2017
*Oryza sativa*	GSE78972	GSM2082863	SRR3209775	Short Day Drought_S7	Leaf	Mar-01-2017
*Oryza sativa*	GSE78972	GSM2082864	SRR3209776	Short Day Drought_S8	Leaf	Mar-01-2017
*Oryza sativa*	GSE78972	GSM2082866	SRR3209778	Long Day Drought_S10	Leaf	Mar-01-2017
*Oryza sativa*	GSE78972	GSM2082868	SRR3209780	Short Day Drought_S12	Leaf	Mar-01-2017
*Oryza sativa*	GSE80811	GSM2137964	SRR3466960	Drought—1 d	Leaves	Feb-14-2017
*Oryza sativa*	GSE80811	GSM2137964	SRR3466961	Drought—1 d	Leaves	Feb-14-2017
*Oryza sativa*	GSE80811	GSM2137965	SRR3466962	Drought—2 d	Leaves	Feb-14-2017
*Oryza sativa*	GSE80811	GSM2137965	SRR3466963	Drought—2 d	Leaves	Feb-14-2017
*Oryza sativa*	GSE80811	GSM2137966	SRR3466964	Drought—3 d	Leaves	Feb-14-2017
*Oryza sativa*	GSE80811	GSM2137966	SRR3466965	Drought—3 d	Leaves	Feb-14-2017
*Oryza sativa*	GSE95668	GSM2520922	SRR5311340	Heat—35 °C—6 h	Leaf	Nov-07-2017
*Oryza sativa*	GSE95668	GSM2520923	SRR5311341	Heat—35 °C—6 h	Leaf	Nov-07-2017
*Zea mays*	GSE71723	GSM1843772	SRR2144414	Drought	Leaf V12	Feb-04-2016
*Zea mays*	GSE71723	GSM1843780	SRR2144422	Drought	Leaf V14	Feb-04-2016
*Zea mays*	GSE71723	GSM1843788	SRR2144430	Drought	Leaf V16	Feb-04-2016
*Zea mays*	GSE71723	GSM1843796	SRR2144438	Drought	Leaf R1	Feb-04-2016
*Zea mays*	GSE71377	GSM1833214	SRR2129983	Drought	Leaf	Jan-22-2016
*Zea mays*	GSE71046	GSM1826061	SRR2106186	wt Salt T7 Rep1	Youngest wrapped leaf	Jan-14-2016
*Zea mays*	GSE71046	GSM1826073	SRR2106198	wt Salt T0 Rep2 + Rep3	Youngest wrapped leaf	Jan-14-2016
*Zea mays*	GSE71046	GSM1826077	SRR2106202	wt Salt T7 Rep2 + Rep3	Youngest wrapped leaf	Jan-14-2016
*Glycine max*	GSE98958	GSM2628302	SRR5569810	Dehydrated	Leaf	May-31-2018
*Glycine max*	GSE98958	GSM2628302	SRR5569811	Dehydrated	Leaf	May-31-2018
*Glycine max*	GSE98958	GSM2628303	SRR5569812	Dehydrated	Leaf	May-31-2018
*Glycine max*	GSE98958	GSM2628303	SRR5569813	Dehydrated	Leaf	May-31-2018
*Glycine max*	GSE69571	GSM1704043	SRR2051086	Salt stress	Leaves	Jul-11-2017
*Glycine max*	GSE69571	GSM1704044	SRR2051087	Salt stress	Leaves	Jul-11-2017
*Glycine max*	GSE69571	GSM1704045	SRR2051088	Salt stress	Leaves	Jul-11-2017
*Glycine max*	GSE69571	GSM1704046	SRR2051089	Salt stress	Leaves	Jul-11-2017
*Glycine max*	GSE70310	GSM1723542	SRR2079645	Drought (15 days)	Leaf r2 stage	Aug-31-2015
*Glycine max*	GSE70310	GSM1723542	SRR2079646	Drought (15 days)	Leaf r2 stage	Aug-31-2015
*Glycine max*	GSE70310	GSM1723542	SRR2079647	Drought (15 days)	Leaf r2 stage	Aug-31-2015
*Glycine max*	GSE69469	GSM1701586	SRR2048167	Drought (3 days ZT0-8 h R1)	Leaves v1 stage	Jul-07-2015
*Glycine max*	GSE69469	GSM1701592	SRR2048173	Drought (3 days ZT4-12 h R1)	Leaves v1 stage	Jul-07-2015
*Glycine max*	GSE69469	GSM1701598	SRR2048179	Drought (3 days ZT8-16 h R1)	Leaves v1 stage	Jul-07-2015
*Glycine max*	GSE69469	GSM1701604	SRR2048185	Drought (3 days ZT12-20 h R1)	Leaves v1 stage	Jul-07-2015
*Glycine max*	GSE69469	GSM1701610	SRR2048191	Drought (3 days ZT16-24 h R1)	Leaves v1 stage	Jul-07-2015
*Glycine max*	GSE69469	GSM1701616	SRR2048197	Drought (3 days ZT20-4 h R1)	Leaves v1 stage	Jul-07-2015

### Analyses

The RNA-seq data was used as input to the *LSTrAP* v1.3 software [14] to construct co-expression networks. Only leaf tissue expression data was used to obtain the networks, to avoid adding noise to the data. LSTrAP was also used to create groups of co-expression, that are clusters of transcripts with correlated expression by using the software MCL version 14–137.

In order to characterize PUFs, *Diamond v0.9.24* [23] was used to align all proteins against the NCBI’s *nr* database (downloaded in January 2018). Diamond BLAST was run with the flag-max-target-seqs 5 and the best hit was selected. *InterproScan* v5.26-65.0 [24] was used to annotate the proteins from the 53 genomes. All other software were run using default parameters. Homolog groups were created using *OrhtoMCL* v2.0.9 [25] and the 53 genome’s proteins as input, with default options.

### Framework interface

The *Machado* software [26] was used to store all data and results, and also provide a web server as an interface for fast data browsing.

### Filter protocols

The *Plantannot* software provides several filters and a text search box that allows searching for molecules by its desired annotation features. These filters are needed to obtain PUFs and to try to relate them to abiotic stresses using RNA-seq expression data and co-expression networks. The Filters menu is separated in 8 fields, of those we are going to use only five: “Organism”, “Feature type”, “Orthology”, “Orthologs_coexpression” and “Analyses”. The “Feature Type” filter has three molecule types, from those the polypeptide box is the only that is going to be always checked and the others blank. By using the other 4 remaining filters, 6 protocols were created (Table 3) as examples of different ways of selecting PUFs. Protocol A [27]: using a lack of both homology and protein domain signatures. Protocol B [28]: using lack of homology, presence of domain signatures—trying to select Domains of Unknown Function (DUF) from PFAM, and the text search “Unknown function”. Protocol C [29]: using homology, lack of protein domain signatures, and the text search “Unknown function”. Protocol D-F [30–32]: same protocols of A–C but using ortholog groups to find homolog proteins with co-expression data related to abiotic stress. The protocols are explained in Table 3.

**Table 3:** Protocols used to characterize PUFs

Name	Objective	Filters (checked boxes only)^a^
Protocol A	Find PUFs from organisms whose proteins are not yet in the NCBI’s “nr” database and have no protein domain signatures found by InterproScan	Analyses: no diamond matches Analyses: no interproscan matches
Protocol B	The same as A but trying to select proteins with the DUF domains from PFAM	Analyses: no diamond matches Analyses: interproscan matches Text search: “Unknown function”
Protocol C	Find PUFs from organisms whose proteins are already public in the “nr” database	Analyses: diamond matches Analyses: no interproscan matches Text search: “Unknown function”
Protocol D	Same as A but using ortholog groups and co-expression networks to relate proteins to abiotic stress	Analyses: no diamond matches Analyses: no interproscan matches Orthology: orthology Orthologs_coexpression: co-expression
Protocol E	Same as B but using ortholog groups and co-expression networks to relate proteins to abiotic stress	Analyses: no diamond matches Analyses: interproscan matches Text search: “Unknown function” Orthology: orthology Orthologs_coexpression: co-expression
Protocol F	Same as C but using ortholog groups and co-expression networks to relate proteins to abiotic stress	Analyses: diamond matches Analyses: no interproscan matches Text search: “Unknown function” Orthology: orthology Orthologs_coexpression: co-expression

^a^For all protocols “Feature type: polypeptide” is always checked

### Overview

**Fig. 1:**
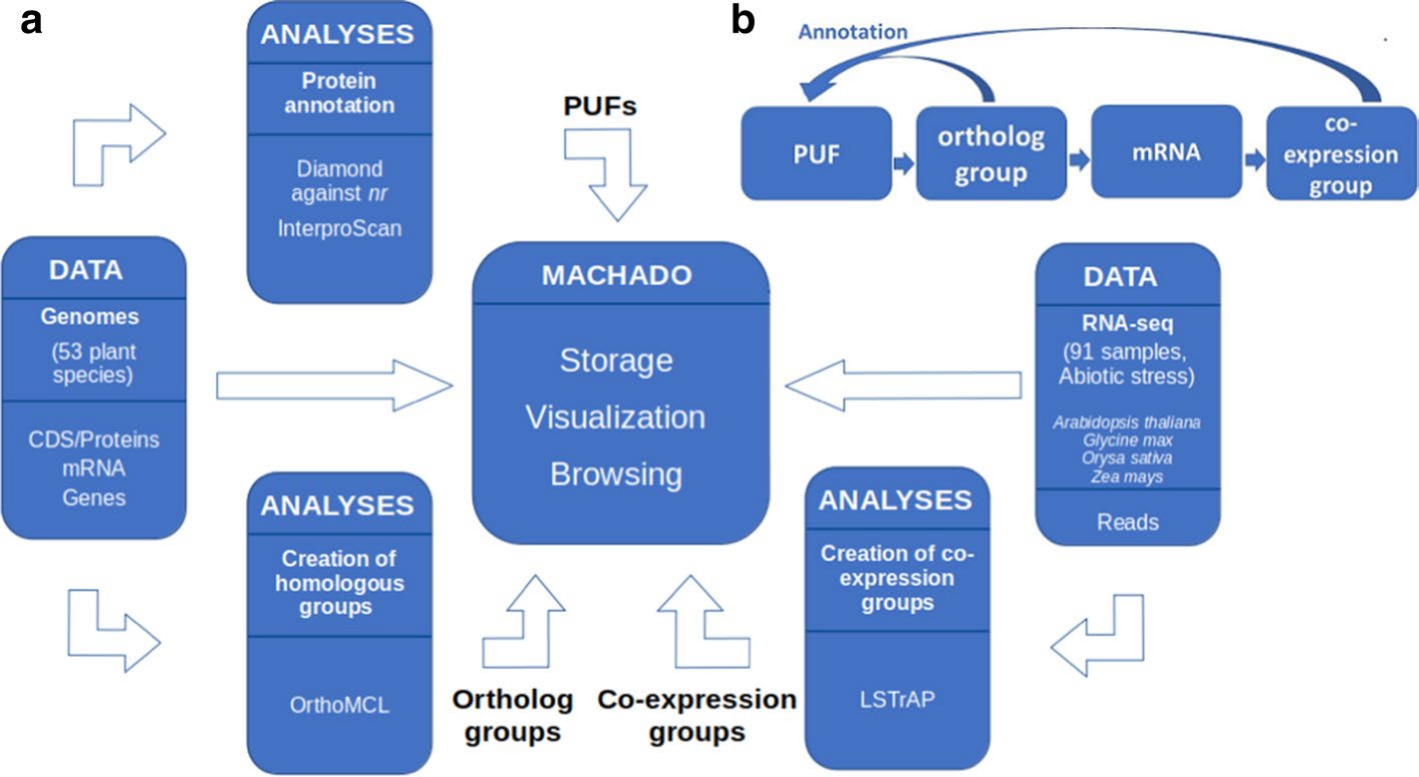
**a** Overview of the Plant Co-expression Annotation Resource processes. **b** Guilt-by-association algorithm used to transfer function annotation to PUFs

An overview of the component processes of the system covering all data and analysis results used as input to the *Machado* framework can be found in Fig. 1a.

### Homolog groups

The 2,332,974 proteins were used as input to the *OrhtoMCL* software to produce 164,267 clusters or groups of homolog proteins (putative orthologs). All groups comprise 1,900,313 proteins, and the mean cluster size was 11.57 protein members, ranging from 1 to 4587 members. It is worth mentioning that 8535 clusters (5.19%) were left with only 1 protein and 75% of all clusters are composed of up to 6 proteins. The ortholog groups are automatically shown in the “Results” frame of the software.

### Co-expression networks

To construct co-expression networks, the 53 GEO Samples (Table 2) were filtered to get expression data only from “leaf” tissue (17, 8, 13, and 15 for Ath, Zma, Gma, and Osa respectively). Four co-expression networks were constructed for each of the four organisms (Ath, Zma, Gma, and Osa), using the default filters and options of LSTrAP. Groups of co-expression were created using the MCL software following the default instructions in LSTrAP. The MCL software clusters the transcripts with correlated expression. Therefore, the groups of co-expression are supposedly correlated to the molecular mechanisms regarding abiotic stress. 524 groups were obtained (169, 36, 177 and 142 for Ath, Zma, Gma and Osa respectively), with mean size of 140, 113, 282 and 225 for Ath, Zma, Gma, and Osa transcript members each, ranging from 1 to 7097 members for Ath, 1 to 4786 for Zma, 1 to 6927 for Gma and 1 to 6636 for Osa.

### PUF characterization

After analyzing all 2,332,974 proteins with *Diamond* and *InterproScan, 72,266* PUFs were characterized (Table 1—Protocol A) as sequences with no annotation using either *Diamond* or *InterproScan*. Another less sensitive way to find PUFs is to text search for “Unknown proteins” and filter for *InterproScan* matches (e.g.: trying to select PFAM’s DUF domains) only or *Diamond* matches only (e.g.: trying to find proteins with uninformative function annotations), which leads to 2409 and 6569 PUFs respectively (Table 1—Protocols B and C respectively).

### PUF annotation

**Fig. 2:**
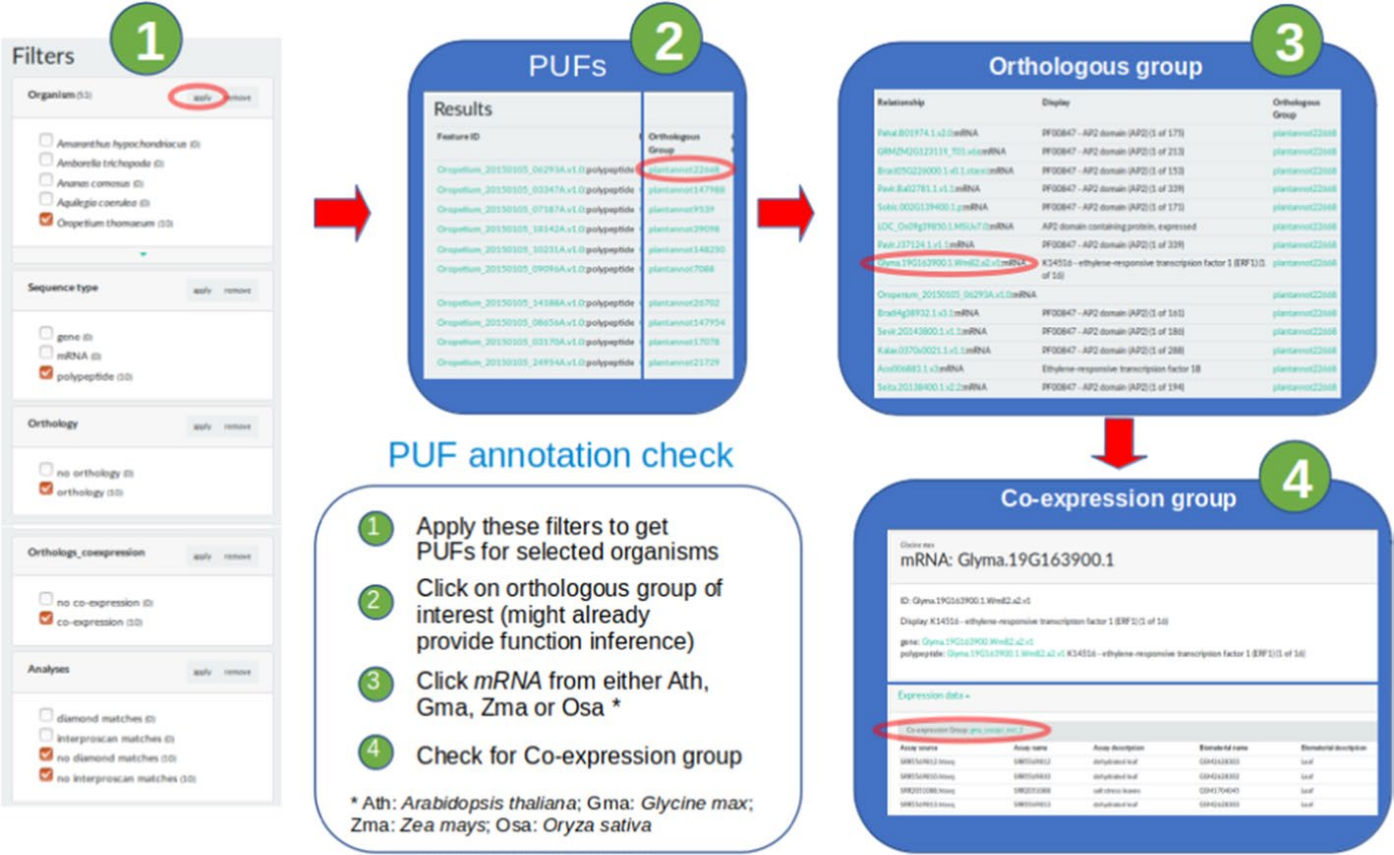
Procotol to check PUF annotation using orthology and co-expression data

As there is no information regarding the function of PUFs, one way to infer function is to link PUFs to other molecules by using orthology groups using a guilt-by-association algorithm (Fig. 1b). Therefore, members from a given ortholog group which already have annotation and/or have protein domains characterized, can be used as a proxy to infer function for the PUF proteins by association. There are 21,895 PUFs as members of ortholog groups which could be a source of functional information and annotation (Protocol A, plus adding the filter “Orthology: orthologs”). Furthermore, whenever a given PUF is part of an ortholog group in which some member, necessarily one of Ath, Gma, Osa, or Zma, have its mRNA composing a co-expression group, then by association, the initial PUF is supposedly also related to response to abiotic stresses in plants by inference (see Fig. 2). 1364 PUFs were related to co-expression groups using filters that were created to automate this selection (Table 3, Protocol D). This method of searching for PUFs was found to be very strict, since it only retrieves proteins that have no annotations whatsoever. However, there are many cases in which PUFs have uninformative annotations, such as: “protein with unknown function”, “putative” or “hypothetical” for example. By modifying Protocol D and text searching for “Unknown function” plus filtering for InterproScan matches only or Diamond matches only, we could annotate 13 and 2,280 PUFs respectively (Table 3, Protocols E and F respectively).

## Utility and discussion

Many web servers and online tools available allow navigation and comparative search of expression and co-expression data in plants. Some tools only work online and are not open source like PLAZA 3.0 [33], others are generic and seek any type of annotation such as CoNeKT [34] or use microarray data like the Genevestigator [35]. *Plantannot* has a very specific role of surveying proteins with unknown function possibly related to abiotic stresses in plants by comparing genomics data of a large number of organisms (53 angiosperm species). Also, the algorithm used to search for PUF annotation includes meta-analyses and data relations that involve searches for similarities of sequences, orthology, and networks of gene co-expression that are specific and unique.

To demonstrate the potential of *Plantannot* we devised 6 protocols for filtering sequences of interest.

From all the 6 protocols, Protocol A was the most permissive, as it seems that most of the organisms have many proteins that do not return as Diamond best hits against the “nr” database. These sequences were selected by the “no diamond matches” filter and could be retrieved (see Table 1). By modifying protocol A and inserting the textual search filter “Unknown function”, led to Protocols B and C.

It is important to mention that genome projects end up having proteins of unknown function annotated in several different ways, by using terms like “hypothetical”, “putative”, “unknown protein”, etc. Therefore, there should be specific text searches for each organism to obtain the best results for selecting PUFs. For example, we needed to adapt the filtering protocols for *Boea hygrometrica,* whose PUFs were best retrieved using the text search “hypothetical”. Other examples can be cited, such as the text search "putative protein" used more efficiently to select PUFs from the organism *Ricinus communis.*

Protocol B uses InterproScan results to search for “Domains of Unknown Function”, or DUFs, from PFAM, which are annotations that could result in more PUFs selected. Protocol C uses the text search to filter Diamond hits and also the original sequence annotations to filter out more PUFs.

The Protocols D-F are more complex protocols that refer to modifications of the Protocols A-C, respectively. They were created by adding filters that could retrieve PUFs that were in the same group of homologous proteins, whose mRNA participate in co-expression network clusters, related do abiotic stresses. This guilt-by-association algorithm explained in Fig. 2 led to filtering of many interesting PUFs that would not be highlighted using protocols A-C, such as those described in the study case section.

Protocol D is quite stringent and after applying it, 15 organisms out of 53 involved did not show any results. The reason for this result is that many organisms already have their proteins deposited in the “nr” database and the Diamond best hits would retrieve their own sequence leading them to be filtered out. This occurred with *Boea hygrometrica* but did not occur with *Oropetium thomaeum,* both described in our case studies above.

Many other protocols can still be created, for example, modifying Protocols D-F filtering only by groups of orthologs (filter “Orthology: orthology”) and not by co-expression. This filter selected 21,895 PUFs that belonged to any group of orthologs. This simpler filter could allow one to infer possible functions to these PUFs by just relating them to the annotations found in the members of their common groups of orthologs. Similarly, after applying Protocol D for all organisms, we could manually curate the 1364 PUFs selected, supposedly related to abiotic stress. By conducting a manual search in the groups of orthologs that these PUFs belong, we were able to confirm 159 PUFs with functions possibly related to abiotic stress, found in annotations of ortholog co-members of these PUFs. This result equals 11.6% of the initial PUFs (check the Additional file 2 for a complete list of PUFs and annotations for all organisms using this methodology).

### Case Study: PUF annotations of desiccation-tolerant species

We used two species known to be tolerant to desiccation as a pilot study for Plantannot as we believe there can be interesting target PUFs related to abiotic stresses to be encountered in these organisms.

### *Oropetium thomaeum*

Recently added to the Phytozome database, *Oropetium thomaeum* [36] is a good candidate to discover genes related to abiotic stress. This grass is resilient to extreme and prolonged drying and must have genes involved in the molecular mechanisms related to the control of this phenotype. To find PUFs for *Oropetium thomaeum* one could use Protocol D as described in Table 1. By doing this one will see 10 PUFs in the “Results” page. As there is no annotation for these proteins (although there is one protein that was already annotated as “PTHR13020:SF36—EXPRESSED PROTEIN (1 of 1” that is not much informative of a function), one can survey the homologous sequences present in the orthologous groups to check for other annotations. In this regard, one can click, for example, on the first member of the “Plantannot22668” group ID, in the “Orthologous Group” column of which the PUF “Oropetium_20150105_06293A.v1.0” is a member. By doing this a new “Results” page will show all members of the “plantannot22668” group. Interestingly the majority of the members are annotated as having an “AP2 domain (PFAM—PF00847)”. By investigating the function of this PFAM domain PF00847, one can discover that AP2 is a transcription factor that has a major role in hormone regulation [37] and one study shows that there is a binding factor DBF1 that binds AP2 and is related to osmotic stress tolerance and abiotic stress responses in *Arabidopsis thaliana* [38]. By association, it is possible to infer that the PUF “Oropetium_20150105_06293A.v1.0” have a function possibly related to “AP2”, and that orthology could be useful to give novel information for the PUFs. Going further, the “Orthologs_coexpression” box checked before, filtered for orthologous groups of which at least one member participates in a co-expression group. Therefore, this adds up more evidence that the PUF “Oropetium_20150105_06293A.v1.0” is a good candidate to be related to abiotic stresses and should be further investigated. To check for the co-expression group related to this PUF, one can follow the procedure in Fig. 2 showing that one member of the ortholog group “Plantannot22668” is a protein from Ath, Osa, Zma or Gma, and whose respective mRNA participate in a co-expression group (in this case, the protein from Gma and its mRNA with the same ID: Glyma.19G163900.1.Wm82.a2.v1). This case study can be performed by checking the tutorial session in *Plantannot*’s initial page.

### *Boea hygrometrica* (*Dorcoceras hygrometricum*)

“Drying without dying” is an essential feature in the evolution of earthly plants and *Boea hygrometrica* is an important model of resurrection plant that survives the drying of its leaves and roots without dying [39]. By using a modified version of Protocol F from Table 3 in which we used the text search word "hypothetical", we recovered 414 PUFs. From these, we obtained possible annotations for 199 PUFs (48% of the total) by surveying the orthologous group members as described above. By manually inspecting all 193 annotations we found that 153 (36.95% of the total) had references to abiotic stresses. From these, we chose 3 interesting PUFs to describe the possible efficiency of our protocol. The first is the protein KZV45975.1, a member of the ortholog group “plantannot11681”, which had members related to “E3 ubiquitin ligase family of proteins”. This family of proteins seems to enhance drought tolerance in *Arabidopsis thaliana* [40]. Another interesting example is the KZV43328.1 protein, a member of “plantannot19415” ortholog group, which has 5 members with the PFAM domain “PF00642—Zinc finger C- × 8-C- × 5-C- × 3-H type (and similar) (zf-CCCH)”. This domain apparently plays roles in abiotic stress response in maize [41]. The final example is the KZV34923.1 protein, who is member of the “plantannot11601” ortholog group which has 17 members that have the PFAM domain “PF05349—GATA-type transcription activator, N-terminal (GATA-N) (1 of 1)”. It is has been shown that GATA like transcription factors are related to abiotic stress responses in rice [42]. It is worth mentioning that some annotations found refer to abiotic stress that were not part of our RNA-seq data set experimental conditions, like resistance to Aluminum and Cadmium. This could be due to the fact that drought and desiccation tolerance involves a complex process to avoid oxidative damage [43] and we speculate if it may share molecular mechanisms with other kinds of abiotic stresses. The full Boea’s PUF survey can be retrieved from the Additional file 1.

### Conclusion

We believe that the Plant Co-expression Annotation Resource can be a valuable bioinformatics tool to be used for the search of proof of concept targets to enter pipelines for the creation of genetic modified crops resistant to abiotic stresses and adapted to climate change.

## Supplementary information

**Additional file 1.** Complete PUF annotation list for* Boea hygrometrica* obtained using a modified version of protocol F.

**Additional file 2.** Complete PUF annotation list for all species using protocol D.

## Data Availability

All datasets used in this article are public and sources cited accordingly. The data that support the findings of this study are available freely from the webserver https://www.machado.cnptia.embrapa.br/plantannot.

## Abbreviations

Ath: *Arabidopsis thaliana*
DUF: Domains of Unknown Function
GM: Genetically Modified
Gma: *Glycine max*
Osa: *Oryza sativa*
PUF: Proteins of Unknown Function
Zma: *Zea mays*
